# Evaluation of Mental Health First Aid training with members of the Vietnamese community in Melbourne, Australia

**DOI:** 10.1186/1752-4458-3-19

**Published:** 2009-09-07

**Authors:** Harry Minas, Erminia Colucci, Anthony F Jorm

**Affiliations:** 1 Centre for International Mental Health, Melbourne School of Population Health, The University of Melbourne, Parkville, Victoria 3010, Australia; 2 Mental Health First Aid Training and Research Program, ORYGEN Youth Health Research Centre, University of Melbourne, Parkville, Victoria 3052, Australia

## Abstract

**Background:**

The aim of this project was to investigate in members of the Vietnamese community in Melbourne the impact of Mental Health First Aid (MHFA) training on attitudes to people with mental illness and on knowledge about mental disorders. Our hypotheses were that at the end of the training participants would have increased knowledge of mental disorders and their treatments, and decreased negative attitudes towards people with mental disorders.

**Methods:**

Respondents were 114 participants in two-day MHFA training workshops for the Vietnamese community in Melbourne conducted by two qualified MHFA trainers. Participants completed the research questionnaire prior to the commencement of the training (pre-test) and at its completion (post-test). The questionnaires assessed negative attitudes towards people with mental illness (as described in four vignettes), ability to recognise the mental disorders described in the vignettes, and knowledge about how to assist someone with one of these disorders. Responses to open-ended questions were content analysed and coded. To evaluate the effect of the training, answers to the structured questions and to the coded open-ended questions given at pre- and post-test were compared using McNemar tests for dichotomous values and Wilcoxon tests for other scores.

**Results:**

Between pre- and post-test there was significant improvement in recognition of mental disorders; more targeted and appropriate mental health first aid responses, and reduction in inappropriate first aid responses; and negative attitudes to the people described in the vignettes declined significantly on many items of the stigma scale.

**Conclusion:**

A two-day, MHFA training course for general members of the Vietnamese community in Melbourne demonstrated significant reductions in stigmatising attitudes, improved knowledge of mental disorders and improved knowledge about appropriate forms of assistance to give to people in the community with mental disorder. There is sufficient evidence to scale up to a population level program for the Vietnamese community, and a need for longitudinal evaluation of such a scaled up program.

## Background

The importance of effective response to mental illness has become increasingly acknowledged as a result of epidemiological data showing the high prevalence of mental disorders in many countries, including Australia [1,2], the large contribution of mental disorders to burden of disease [3], the high economic and social costs of mental disorders [4] and evidence of the personal, social and economic benefits of good mental health [5]. In Australia and elsewhere these realizations have given impetus to a sustained program of mental health system reform [6-8] with the intention of improving population mental health and the performance of mental health systems that have struggled to keep pace with community needs [9]. There is an emerging consensus that several elements are essential for successful reform and for the development of mental health services that are effective, affordable, accessible and equitable. Among these are a population mental health perspective, a whole of government approach [10,11], genuine stakeholder participation in decision-making [12,13], and an informed and engaged community [14,15].

### The Vietnamese community in Australia

In 2006 there were 180,400 Vietnam-born persons in Australia (0.88% of the Australian population) of whom 60,395 lived in Victoria (1.15% of the population), mostly in Melbourne [16]. Studies of mental health of Vietnam-born community members in Australia have revealed inconsistent findings on prevalence of mental disorder. The prevalence of mental disorder among Vietnamese children and adolescents in Perth, Australia, was broadly similar to that in the Australia-born population [17], with the additional finding that parents substantially under-reported mental disorder in their children. In a large epidemiological study of Vietnam-born adults in Sydney, Australia, Silove and colleagues [18] reported substantially lower prevalence of mental disorder (6.9%) than that found for all Australians (18.6%). in the National Mental Health Survey [19]. Despite the fact that the Vietnam-born had had substantially greater exposure to traumatic events than the Australia-born, the rate of PTSD in both populations was 3.5%

There are consistent data that Vietnam-born people in Australia make use of mental health services at substantially lower rates than do the Australia-born [20,21]. In the Silove et al study [18] the Vietnam-born sought help from mental health professionals at much lower rates than did the Australia-born. A study of access to public mental health services in Victoria, Australia, showed that the Vietnam-born accessed such services at substantially lower rates than the Australia-born [20]. Wagner et al [22] found that Vietnamese patients who had attended an anxiety disorders clinic in Sydney were more likely to drop out of treatment. Wagner et al further observed that a sample of Vietnamese people in the community did not differentiate clearly between the terms 'stress', 'anxiety' and 'depression'. Additionally, many participants felt that there was a generally negative cultural attitude towards people suffering from these problems and towards the mental health system.

Phan [23] conducted interviews with 324 Vietnamese-speaking adult caregivers living in Sydney, focusing on types of services used for identifying and/or intervening for *binh tam than *(mental ill-health), difficulties encountered, and recommendations for enhancing services. Almost one in two interviewees had used such services during the previous twelve months, including those provided by local Vietnamese-speaking doctors, Asian naturalists, spiritual healers, herbalists, and folk healers, as well as mainstream psychiatric hospital facilities and community services.

### Mental Health First Aid

While general community members often have some knowledge about common physical health problems, and what to do when they occur, knowledge about mental health problems is much less well developed [24]. First aid for common physical health problems and for medical emergencies is well established and large numbers of people in the general community receive appropriate first aid training. Lack of knowledge about mental disorders contributes to stigma, inhibits appropriate and timely help-seeking [25] and results in a less than adequate range of first aid responses to people with mental disorders [26]. In response to these problems the MHFA training program was developed [27] on the basis of the expectation that people with mental health problems can potentially be assisted by those in their social network [28] and that a suitable training program for members of the general public would improve the necessary confidence and skills to provide basic help [29]. The MHFA program [30], which has now been implemented in many countries, aims to widen the base of people with the knowledge and skills to provide basic assistance to people in the community with mental health problems and in the early stages of a mental health crisis. The 12-hour training course [25] gives an overview of the major categories of mental health problems, introduces an MHFA Action Plan and applies those actions to problems of depression, anxiety disorders, psychosis and substance use disorder [30,31]. The course also covers the following mental health crisis situations: how to help a suicidal person, a person having a panic attack, a person who has experienced a traumatic event, a person with psychosis who is perceived to be threatening and a person who has overdosed.

The MHFA program has more than 1,000 instructors delivering training across Australia and there are organisations in twelve countries that have adapted the MHFA Australia program for local use. The training course has been evaluated in various settings, with different samples using a range of methods [27,32-34]. A review of evaluations of MHFA training [25] has highlighted consistent positive benefits in knowledge, behaviour, intentions and attitudes in participants.

Although the benefits of MHFA have been demonstrated in several studies there has, as yet, been no evaluation of training carried out in an immigrant community. The aim of this project was to investigate in members of the Vietnamese community in Melbourne the impact of MHFA training on attitudes to people with mental illness, and on knowledge about mental disorders and knowledge about appropriate first aid responses. Our hypotheses were that at the end of the training participants would have increased knowledge of mental disorders and appropriate mental health first aid responses, and decreased negative attitudes towards people with mental disorders.

## Methods

The 12-hour, two-day MHFA training program was advertised widely through community channels in the Vietnamese community in Melbourne, emphasising that all community members were welcome to participate in the program, which was delivered free of charge. Participants in the training programs were general members of the community who registered for the training. There was no specific selection process. An interest in learning about mental health was sufficient. Participants in three training groups were invited by the trainers to participate in the evaluation of the program by anonymously completing the evaluation questionnaire prior to the commencement of the training (pre-test) and at its completion (post-test).

The training was delivered by two qualified MHFA instructors, both mental health professionals (psychology and social work), and both born in Vietnam, who were involved in the cultural adaptation of the MHFA training course and manual. The adapted MHFA manual was translated into Vietnamese by a Vietnamese psychiatrist. The training program was conducted in Vietnamese.

The evaluation questionnaire consisted of the following components:

1) A section seeking brief socio-demographic information.

2) Presentation of four brief vignettes about each of which the following questions were asked:

a) What would you say, if anything, is wrong with John?

b) Imagine John is someone you have known for a long time and care about. You want to help him. What would you do?

c) Has anyone in your family or close circle of friends ever had problems similar to John's?

d) Have they received any professional help or treatment for these problems?'

e) Have you ever had a job that involved providing treatment or services to a person with a problem like John's?

The vignettes were taken from a paper by Griffiths et al. [35] reporting a study of stigma associated with mental disorders. The vignettes described a person with: depression; depression with suicidal ideation; early schizophrenia; and chronic schizophrenia. Each of the disorders depicted in the vignettes satisfied both DSM-IV and ICD-10 diagnostic criteria for either major depressive disorder or schizophrenia.

3) Following the open-ended responses to the questions above, respondents were asked to indicate level of agreement with a number of statements in relation to each vignette on a 5-point scale (1 = Strongly agree, 2 = Agree, 3 = Neither agree nor disagree, 4 = Disagree, 5 = Strongly disagree). The first group of statements asked respondents to "indicate how strongly you agree or disagree with each of the following statements by ticking the appropriate box". This was intended as a measure of personal stigma [35]. The second groups of statements, identical in content to the first, asked respondents to "indicate what you think *most other people *believe". This was intended as a measure of perceived stigma [35]. The statements concerning which participants were asked to indicate level of agreement were:

a) People with a problem like John's could snap out of it if they wanted

b) A problem like John's is a sign of personal weakness

c) A problem like John's is not a real medical illness

d) People with a problem like John's are dangerous

e) It is best to avoid people with a problem like John's so you don't develop the problem yourself

f) People with a problem like John's are unpredictable

g) If I had a problem like John's I would not tell anyone

h) I would not employ someone if I knew they had a problem like John's

i) I would not vote for a politician if I knew they had a problem like John's

The post-test questionnaire was identical except that the socio-demographic information was excluded. The questionnaires were presented in English, but participants were invited to write their responses to the open-ended questions in either English or Vietnamese. Vietnamese responses were translated into English by a bilingual mental health professional.

The data collected were analysed in two ways. First, data collected at pre-test were analysed to examine participants' recognition of disorders, their mental health first aid responses and the level of personal and perceived stigma. The data collected at post-test were compared with data collected at pre-test to measure change in recognition of disorders, first aid responses and stigma towards people with mental disorders. Only participants who completed both the pre- and post-test questionnaires were included for analysis. From a total of 138 training participants, 114 returned completed pre- and post-test questionnaires (82.6% response rate). Twenty-four questionnaires were excluded because of incomplete data or because the code that was necessary to link pre- and post-test questionnaires was not entered.

For each vignette there were two open-ended questions. (1. What would you say, if anything, is wrong with John? 2. Imagine John is someone you have known for a long time and care about. You want to help him. What would you do?) The first was to assess participants' recognition of the mental disorder described in the vignette and the second inquired about mental health first aid responses. The four diagnoses that were considered correct were: depression (for vignette 1), psychosis or schizophrenia (vignettes 2 and 4) and depression and/or a reference to suicidality (vignette 3). Responses were coded 0 (incorrect diagnosis) or 1 (correct).

In the MHFA training program a structured response, consisting of five actions, is taught. The actions are:

**1) A**ssess risk of suicide or harm

**2) L**isten non-judgmentally

**3) G**ive reassurance and information

**4) E**ncourage the person to get appropriate professional help

**5) E**ncourage self-help strategies

The initial letters of these actions constitute the mnemonic ALGEE.

The free responses to the second question (What would you do?) were coded on a 0-2 scale according to the quality of the response for each of the ALGEE actions: 0 = no mention or inadequate response, 1 = superficial response, 2 = specific details. The ratings were then summed to give a total score out of 10. Detailed scoring criteria were drawn up for this purpose. A research assistant was trained in the use of the scoring criteria and she rated the responses after their order had been randomized. Randomization of the order ensured that the rater was not told what vignette the response was to, nor whether it was a pre-test or post-test response. In order to assess the reliability of her ratings, the research assistant was also asked to score 40 responses from another data set that had been previously scored using the consensus of four experts in MHFA. Her ratings correlated highly with the expert consensus ratings. Pearson correlations were: A 1.00, L 0.90, G 0.78, E (Professional) 0.81, E (Self-help) 0.87, Total 0.95.

To evaluate the effect of the training, answers to the structured questions and to the coded open-ended questions given at pre- and post-test were compared using McNemar tests for dichotomous values and Wilcoxon tests for other scores. These non-parametric tests were used because the scores did not meet the distributional assumptions of parametric tests. The analysis was carried out using SPSS 16.0. The p < 0.05 significance level was used. Mean scores, standard deviations and p-values are reported in the tables below.

## Results

### Participant characteristics

The sample (Table 1) consisted of more women than men, had a mean age of 37.8 years (SD 14.5, range 18-69), participants were well educated (almost half had a University degree and more than a third were students at the time of the training), and most (77.2%) were born in Vietnam.

**Table 1:** Socio-demographic characteristics of participants

Female	67.9%
Age group	
18-39	51.8%
40-59	41.1%
60+	7.1%

Country of birth	
Vietnam	77.2%
Australia	17.5%
Other	5.3%

Mean age at arrival in Australia	16.43 yrs (SD 7.66)

University degree	48.6%

Employed	55.9%

Students	37.8%

### Previous personal and/or professional contacts

A substantial proportion of participants (between 15.8% and 57.9%) reported having had contact with people with problems similar to those described in the vignettes (Table 2). Participants also reported that, on average, only a quarter of the people they had had contact with had received any professional help for their problems. While approximately a third of participants were employed in a position that involved "providing treatment or services" to a person with a problem like the one in the vignette, these services were most commonly social, employment or housing services. There were no participants who were mental health professionals or who were providing specific mental health treatment or other specialised mental health services.

**Table 2:** Participants who had family or friends who had experienced problems similar to vignettes, proportion of contacts who had sought help, and proportion of participants who had been employed in a position that involved providing treatment or services

	**Vignette**			
	**Depression** **%**	**Early schizophrenia** **%**	**Depression with suicidal thoughts** **%**	**Chronic schizophrenia** **%**

A member of participant's family or close circle of friends has had a problem similar to vignette description	57.9	33.6	36.1	15.8

Proportion of people with problem who had sought help	35.1	24.8	21.3	14.9

Participant has worked in a position that involved providing treatment or services to a person with a problem like...	43.4	34.8	31.2	29.3

### Change in recognition of disorders

Table 3 shows that there was a highly significant (p < 0.001) improvement between pre- and post-test in the ability of participants to recognise the disorders described in three of the four vignettes. For the depression with suicidal thoughts vignette, there was an improvement, but this was not statistically significant (p = 0.082)

**Table 3:** Percentage with correct recognition of disorder in vignettes before and after training course

**Vignette**	**Pre-test** **%**	**Post-test** **%**
Depression*	53.5	85.2
Early schizophrenia*	.40.6	66.3
Depression with suicidal thoughts	68.3	79.2
Chronic schizophrenia*	28.7	69.3

* p < 0.001

By teaching participants the psychiatric terms for these disorders, MHFA training also eliminated the use of the stigmatizing language that was at times used in the pre-test questionnaire (e.g. "Yes, he's crazy" (#64); "John is mad seriously" (#71); "He has something wrong in his brain. He starts to be mental" (#86)).

### Change in Mental Health First Aid responses

Table 4 shows the means (and SDs) of each category of first aid response at pre- and post-test for each of the vignettes. Wicoxon tests were statistically significant for all elements of the ALGEE action plan, except for encouraging the person to seek professional help. However, for this action, the scores were high at pre-test, allowing less room for improvement. Pre- and post-training responses of participants illustrate the kinds of improvement that occurred (Table 5).

**Table 4:** Means (and SDs) of first aid responses for each vignette

**First aid responses**	**Depression**		**Early schizophrenia**		**Depression with suicidal thoughts**		**Chronic schizophrenia**	
	**T1**	**T2**	**T1**	**T2**	**T1**	**T2**	**T1**	**T2**

**A**ssess risk of suicide or harm	0.02 (0.19)	0.79*** (0.89)	0.02 (0.19)	0.71*** (0.82)	0.29 (0.71)	1.16*** (0.86)	0.04 (0.20)	0.67*** (0.75)

**L**isten non-judgmentally	0.60 (0.64)	1.25*** (0.70)	0.47 (0.65)	.1.15*** (0.75)	0.53 (0.62)	0.99 *** (0.74)	0.32 (0.59)	0.98*** (0.77)

**G**ive reassurance and information	0.25 (0.45)	0.98*** (0.81)	0.20 (0.42)	0.88*** (0.77)	0.28 (0.49)	.0.80*** (0.79)	0.15 (0.39)	0.69*** (0.72)

**E**ncourage the person to get appropriate professional help	1.12 (0.94)	1.22 (0.71)	1.34 (0.88)	1.27 (0.66)	1.25 (0.87)	1.20 (0.69)	1.38 (0.80)	1.23 (0.61)

**E**ncourage self-help strategies	0.16 (0.42)	0.77*** (0.64)	0.22 (0.50)	0.55*** (0.61)	0.21 (0.46)	0.54*** (0.59)	0.16 (0.44)	.0.40** (0.51)

**ALGEE **(Total)	2.15 (1.11)	5.02*** (2.47)	2.25 (1.04)	4.55*** (2.32)	2.56 (1.41)	4.69*** (2.30)	2.04 (1.15)	3.96*** (2.26)

** p < 0.001

*** p < 0.001

**Table 5:** Examples illustrating improvements in mental health first aid responses from pre- to post-test

**Pre-test response**	**Post-test response**
**Participant #5, Female, Depression vignette**	

Talk to him, let him know that you're concerned and that you'd like to help out some way	Assess risk of suicide/harm - talk to him and find out whether he has been having suicidal thoughts. Better to ask directly than not to. Listen non-judgmentally - don't give advice or tell him to cheer up, acknowledge he is feeling down. Give reassurance/info. Encourage him to seek professional help (perhaps go with him to a counsellor). Encourage self-help strategies - perhaps buy him a book? [ALGEE].

**Participant #5, Female, Chronic schizophrenia vignette**	

Perhaps take him to a professional? I couldn't deal with it by myself, but I agree he needs help.	Assess the risk of suicide/self-harm. Talk to Tim and try to gather as much information about his delusions and hallucinations whilst listening non judgmentally. Acknowledge that they are real for Tim but not for you. Give reassurance and information. Let him know that he can be safe with some professional help and that you'd like to help. Comply with any reasonable requests, don't joke about his delusions because they're very real to him. Keep him in your trust. Make him feel comfortable in your presence. Seek professional help.

**Participant #33, Female, Chronic schizophrenia vignette**	

Come to his place and find out find out what has happened before Peter behaved that way; talk to Peter as well, see if he is able to explain/express what he is feeling	See if he is at risk to himself/others; talk to him, listen non-judgementally; encourage to seek some professional care

**Participant #36, Female, Early schizophrenia vignette**	

I will talk to him and encourage him to see a specialist but I won't let him know the specialist is a psychiatrist. I will have to use his imagination to create a story to make him feel I have the same problem. We will go to see the police or CIA, etc; summary: talk along with his imagination	Encourage to see appropriate medical practitioner; to be with him or asking someone to be with him; talk to him.

**Participant #56, Male, Depression with suicidal thoughts vignette**	

I would advise him should be go out for relay as the beach.	Have conversation with John to find & assess risk of suicidality, listen, give assurance, encourage John to see a specialist or find out who could provide effective treatment

**Participant #101, Male, Depression vignette**	

Strongly recommend Mark to see family doctor; refer Mark to local Mental Health service (even against Mark's will);	I will follow 5 steps of Mental Health first aid: a) assessing Mark's suicide risk; b) listening to Mark without judging, make sure someone stays wit Mark to protect his safety; c) reassuring Mark and provide information to Mark; d) recommending Mark to seekhelp fromGP or from local mental Health service, refer Mark to local crisis act team; e)continue to support Mark and his parents

### Change in attitudes

Table 6 shows participants' level of disagreement with negative attitudes towards people with mental disorders at pre- and post-test assessments for each item. MHFA training had an impact on participants' negative attitudes towards mental disorders. In particular, the training seemed to reduce participants' beliefs that a mental disorder is a sign of personal weakness and that it is not a real illness.

**Table 6:** Means (and SDs) for the attitudes items for each vignette

**Item**	**Depression**		**Early schizophrenia**		**Depression with suicidal thoughts**		**Chronic schizophrenia**	
	**T1**	**T2**	**T1**	**T2**	**T1**	**T2**	**T1**	**T2**

People with a problem like John's could snap out of they wanted	3.49 (1.15)	3.62 (1.20)	3.67 (1.26)	3.99** (1.14)	3.61 (1.24)	3.88* (1.12)	3.76 (1.30)	3.90 (1.27)

A problem like John's is a sign of personal weakness	3.36 (1.25)	3.59* (1.28)	3.40 (1.31)	3.69** (1.26)	3.33 (1.26)	3.63** (1.22)	3.49 (1.33)	3.77* (1.26)

A problem like John's is not a real medical illness	3.61 (1.01)	3.89** (.97)	3.87 (1.10)	4.12* (.97)	3.83 (1.11)	3.88 (1.04)	3.75 (1.27)	4.15** (1.04)

People with a problem like John's are dangerous	3.50 (1.20)	3.83** (1.02)	2.98 (1.08)	3.15 (1.08)	2.87 (1.24)	3.29** (1.24)	2.92 (1.18)	3.06 (1.24)

It is best to avoid people with a problem like John's so you don't develop the problem yourself	4.23 (.95)	4.24 (.90)	4.09 (.92)	4.13 (.88)	4.01 (.96)	4.04 (.98)	4.13 (.90)	3.95 (.98)

People with a problem like John's are unpredictable	2.99 (1.10)	3.40*** (1.08)	2.67 (1.05)	2.93* (1.10)	2.83 (1.18)	2.99 (1.20)	2.70 (1.22)	2.74 (1.25)

If I had a problem like John's I would not tell anyone	3.82 (1.00)	4.02* (.91)	3.63 (1.09)	3.82 (1.06)	3.61 (1.19)	3.89* (1.03)	3.45 (1.21)	3.76* (1.22)

I would not employ someone if I knew they had a problem like John's	3.19 (.98)	3.30 (.98)	2.70 (.99)	2.69 (1.03)	2.72 (1.08)	2.81 (1.12)	2.51 (1.06)	2.54 (1.78)

I would not vote for a politician if I knew they had a problem like John's	2.68 (1.12)	2.90* (1.15)	2.30 (.95)	2.35 (1.03)	2.53 (1.02)	2.46 (1.16)	2.29 (1.08)	2.33 (1.13)

**Note**: Higher scores indicate higher disagreement with the statement - less stigmatising attitude. Asterisks represent the levels of significance: * for p < .05, ** for p < .01, *** for p < .001)

As well as assessing *personal stigma *towards people with mental illness, we investigated *perceived stigma*, i.e. participants' views about the probable attitudes of the general community towards people with mental illness. As expected, there were no substantial changes in participants' perception of other people's attitudes towards people with mental disorders and only a few items showed a statistically significant difference between pre- and post-test. The only consistent finding was a reduction in belief that other people would conceal that they had a problem like the ones in the schizophrenia vignettes.

## Discussion

Although this is the first evaluation of MHFA training with an immigrant group in a language other than English, and the results should be regarded as preliminary, they are encouraging. The findings confirmed our hypotheses, that there would be improved knowledge of mental disorders, reduced negative attitudes to persons with mental disorders and improved knowledge concerning appropriate early response to a person.

Vietnam-born members of the Australian community use public mental health services at substantially lower rates than do the Australia-born [20]. This is true also for a large number of other immigrant and refugee communities in Australia and in other countries of immigration. Many possible explanations have been advanced for this consistent observation, including low mental health literacy in immigrant communities, high levels of stigma attaching to mental illness and to seeking hep from mental health services, lack of knowledge about how to gain access to mental health services, difficulties in communication with mental health services, and many others [36-38]. It would appear from the findings reported here that, through the means of a brief and inexpensive training course that is understandable to general members of the Vietnamese community, it is possible to improve knowledge concerning mental disorders, to teach more appropriate responses when mental disorder is present, and to reduce negative and stigmatising attitudes towards mental illness and people with mental illness. This is the first study to demonstrate the value of MHFA training, suitably modified by skilled mental health professionals who are familiar with the relevant cultural issues, with a non-English speaking immigrant community.

The findings from this study are similar to the conclusions drawn from a review of evaluation studies of the immediate impact of MHFA training [25]. However, we do not know whether the changes observed during the course of the training will be sustained, whether the new knowledge and skills will be used appropriately (or at all), and whether assisting in the recommended manner will actually bring benefit to people with mental illness with whom the mental health first aiders come into contact. Reports from evaluations carried out with English speaking populations show sustained benefits to training participants 5-6 months post-training and continued improved confidence in offering assistance. We do not know whether this will be the case in immigrant communities. These are questions awaiting further studies.

The body of evidence that has accumulated about the benefits of MHFA training in English-speaking communities, and the entirely consonant findings from this study in the Vietnamese community, offers a rationale for substantially scaling up such a training endeavour with the Vietnamese community so that it might have a population level impact. It will be necessary to evaluate, using longitudinal studies, whether such a population level program results in improvement in general community mental literacy, reduction in stigma towards people with mental illness, seeking psychiatric treatment and care when it is required, greater demand for and better access to public mental health services, earlier presentation (early in the episode of illness and at an early age - given the findings of under-detection of mental health problems in Vietnamese children and adolescents by their parents [17]), and improved Vietnamese community mental health.

Because Mental Health Matters: Victorian Mental Health Reform Strategy 2009-2019 [11] sets out the major strategic directions for Victorian mental health services over the next 10 years. The strategy pays particular attention to the need to consider cultural and linguistic diversity in developing more effective and appropriate mental health services. ("Reform also requires concerted change in the culture of service delivery to achieve a more welcoming, participatory service environment that is sensitive to diversity." Page 10). A rigorously evaluated program of MHFA training for the Vietnamese community at a scale that could have population level impacts would be fully consistent with each of the six guiding principles of the new Victorian mental health strategy, which are:

• Consumer-centred service provision

• Family and carer inclusion

• Population-based planning

• Social model of health

• Equity and responsiveness to diversity

• Evidence-based practice

Such a population level program, which engages and enables members of the community to contribute directly to community mental health, would contribute to the core elements of the strategy - prevention, early intervention, a focus on recovery, and social inclusion.

## Conclusion

MHFA training has been shown to be effective in the Vietnamese community in Melbourne in improving knowledge about mental disorders and knowledge about appropriate helping responses, and in reducing negative stigmatising attitudes towards people with mental illness. The evidence from this study, together with the accumulated evidence of the benefits of MHFA training in the general Australian community, suggests that this approach should be scaled up to a level where it can have an impact on the whole of the Vietnamese community in Melbourne. Such a scaled up population mental health program should be evaluated over time and, if the evidence supports such extension, should be extended to other immigrant and refugee communities that are under-represented in Victorian public mental health services.

## Competing interests

The authors declare that they have no competing interests.

## Authors' contributions

HM conceived the study and arranged for it to be done, constructed the questionnaires, participated in discussions concerning analyses and wrote the final version of the paper. EC carried out the data analyses, wrote a first draft of the paper and contributed to the final version of the paper. AFJ contributed the questionnaire content, data analysis and editing of the manuscript. All authors have read and approved the final manuscript.
